# RON12, a novel *Plasmodium*-specific rhoptry neck protein important for parasite proliferation

**DOI:** 10.1111/cmi.12181

**Published:** 2013-08-28

**Authors:** Ellen Knuepfer, Oniz Suleyman, Anton R Dluzewski, Ursula Straschil, Aisling H O'Keeffe, Solabomi A Ogun, Judith L Green, Munira Grainger, Rita Tewari, Anthony A Holder

**Affiliations:** 1 Division of Parasitology, MRC National Institute for Medical ResearchMill Hill, London, NW7 1AA, UK; 2 Division of Cell and Molecular Biology, Imperial College of Science, Technology and MedicineLondon, SW7 2AZ, UK; 3 Centre for Genetics and Genomics, School of Biology, Queens Medical Centre, University of NottinghamNottingham, NG2 7UH, UK

## Abstract

Apicomplexan parasites invade host cells by a conserved mechanism: parasite proteins are secreted from apical organelles, anchored in the host cell plasma membrane, and then interact with integral membrane proteins on the zoite surface to form the moving junction (MJ). The junction moves from the anterior to the posterior of the parasite resulting in parasite internalization into the host cell within a parasitophorous vacuole (PV). Conserved as well as coccidia-unique rhoptry neck proteins (RONs) have been described, some of which associate with the MJ. Here we report a novel RON, which we call RON12. RON12 is found only in *Plasmodium* and is highly conserved across the genus. RON12 lacks a membrane anchor and is a major soluble component of the nascent PV. The bulk of RON12 secretion happens late during invasion (after parasite internalization) allowing accumulation in the fully formed PV with a small proportion of RON12 also apparent occasionally in structures resembling the MJ. RON12, unlike most other RONs is not essential, but deletion of the gene does affect parasite proliferation. The data suggest that although the overall mechanism of invasion by Apicomplexanparasites is conserved, additional components depending on the parasite–host cell combination are required.

## Introduction

The Apicomplexa is a protozoan phylum containing thousands of mostly obligate intracellular parasites. Among these are a number of aetiological agents of medical and veterinary importance, including *Plasmodium, Toxoplasma, Babesia, Neospora, Cryptosporidium, Eimeria* and *Theileria*. Host cell invasion is paramount to the zoite stages, as these can only live for short periods of time as extracellular forms. Although the host cell range of Apicomplexan parasites is vast, the mechanism of cell invasion is largely conserved (Besteiro *et al*., 2011).

Detailed steps of invasion have been best described in *Plasmodium* and *Toxoplasma* (Sibley, 2011; Cowman *et al*., 2012). It is believed that parasites attach to host cells using low-affinity interactions between parasite plasma membrane-anchored surface proteins and host cell receptors. This initial attachment has been visualized in the case of *Plasmodium* and *Babesia* merozoites invading erythrocytes, as dramatic movement of the zoite over the host cell resulting in frequent and extensive deformation of the erythrocyte (Gilson and Crabb, 2009; Asada *et al*., 2012). Initial attachment is followed by reorientation of the highly polarized zoite, anterior end first, towards the host cell. The reorientation process is not believed to be an active process but rather a consequence of a gradient of adhesive proteins towards the anterior end of the parasite (Mitchell *et al*., 2004; Sanders *et al*., 2005). AMA1 is thought to be one of these adhesive proteins residing in the phylum-defining apical secretory organelles (micronemes) which are released on to the merozoite surface upon egress. Invasion proceeds by formation of the moving junction (MJ), a structure identified 30 years ago by electron microscopy as an electron dense interface between parasite and host cell that constitutes an aperture through which the parasite enters into the host cell (Aikawa *et al*., 1978). The MJ is believed to be a fulcrum for the parasite's driving force during invasion but also a sieve selecting which host proteins are allowed into the developing parasitophorous vacuolar membrane (PVM). In recent years the molecular composition of the MJ has been elucidated: the parasite translocates its own proteins from the rhoptry neck (RON2, 4, 5 and *Toxoplasma gondii* [Tg]RON8) into the host cell during invasion to form a solid connection not only with the host cell cytoskeleton but also with the micronemal transmembrane protein, apical membrane antigen 1 (AMA1) which is released onto the zoite surface and binds directly to RON2 located within the host cell's plasmalemma (Alexander *et al*., 2005; Lebrun *et al*., 2005; Besteiro *et al*., 2009; Lamarque *et al*., 2011; Straub *et al*., 2011; Tyler and Boothroyd, 2011). During invasion the MJ progresses backwards over the zoite surface, the rhoptry bulb contents are secreted forming the PVM, then the zoite completes entry into the host cell and both the host plasmalemma and the PVM seal and separate, a process nicely visualized by Riglar and colleagues (2011). Besides being structural parts of the MJ, the interaction of AMA1 on the parasite surface with RON2 which has been translocated to the host cell plasma membrane might provide one of potentially multiple sensing checkpoints that results in rhoptry secretion (Richard *et al*., 2010; Srinivasan *et al*., 2011). Alternatively, the trigger for rhoptry secretion may be activated by micronemal proteins relocated to the parasite surface binding to their host cell receptors (such as the EBA175 – glycophorin A interaction) (Singh *et al*., 2010). The newly invaded parasite now resides in the host cell within its own niche of the PV where it feeds and replicates before egress and another cycle of host cell invasion.

Invasion is believed to be an active process based on motility of the parasite. When cytochalasin D, a well-known mycotoxin inhibitor of actin filament polymerization is added to *Plasmodium* parasites, merozoites can still egress, attach to the host cell and reorientate ready for invasion. A moving junction is still able to form and rhoptry secretion is not affected in the presence of cytochalasin D but merozoites fail to actively invade, remaining attached to the surface of the erythrocyte (Miller *et al*., 1979). The actin myosin motor localizes to the subalveolar space, with actin attached via aldolase to the cytoplasmic tails of invasion molecules and myosin A anchored via a protein complex in the inner membrane complex (IMC) (Daher and Soldati-Favre, 2009). Curiously, on visualizing filamentous actin during invasion, the ring of actin outlines the posterior edge of the MJ and does not colocalize with RON4, the MJ marker, suggesting that the tight interaction between the secreted micronemal adhesion molecules and the host cell receptors occurs posterior to and adjacent to the MJ (Angrisano *et al*., 2012). In another study the role of AMA1 as the principal parasite surface molecule involved in formation of the moving junction was called into question, with both *Plasmodium* sporozoites and *Toxoplasma* tachyzoites forming fully functional MJs containing RON4 in conditional knockout clones of AMA1 (Giovannini *et al*., 2011). Hence, despite a sophisticated knowledge of the intricate stages of invasion and molecules involved in this process, which might represent excellent intervention candidates, further details of the function of known players as well as identification of novel molecules involved in this process need to be uncovered.

Here we describe the identification of a novel rhoptry neck protein, RON12, which is mostly retained within the rhoptry neck until completion of invasion, before being secreted into the nascent PV. RON12 was also detected at the MJ in a small number of invasion events suggesting that it might also play a direct role in host cell invasion. RON12 is the first described *Plasmodium*-specific rhoptry neck protein which is not essential for *in vitro* parasite growth but is nevertheless important in parasite proliferation.

## Results

### Identification of a novel rhoptry neck protein

**Figure 1:**
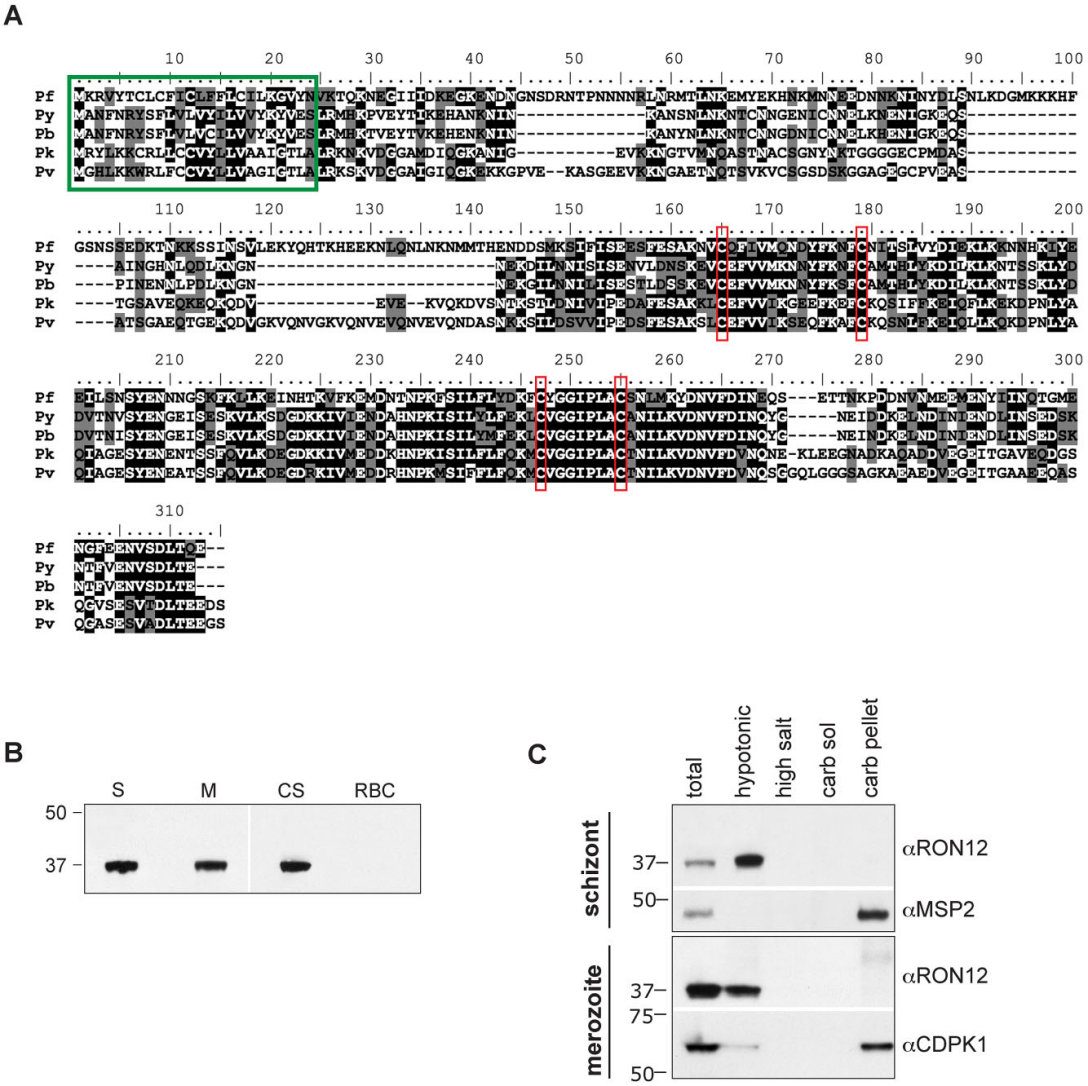
Identification of RON 12, a novel *Plasmodium*-specific protein expressed in blood-stage parasites.A. clustalw alignment of RON12 sequences from *P. falciparum* (Pf), *P. yoelii* (Py), *P. berghei* (Pb), *P. knowlesi* (Pk) and *P. vivax* (Pv). Identical residues are highlighted in black, residues conserved in three or more species are highlighted in grey. Green box indicates putative signal peptides; conserved cysteines are boxed in red.B. Immunoblot of purified schizont (S), merozoite (M), uninfected erythrocyte (RBC) extracts and culture supernatant (CS) probed with RON12-specific antibodies.C. Analysis of protein solubility: schizonts and merozoites were serially extracted in hypotonic, high-salt or -carbonate buffer [resulting in membrane-associated (carb sol) or integral membrane protein fraction (carb pellet)] followed by SDS-PAGE and immunoblotting with the antibodies indicated. Total parasite lysate was used as immunoblotting control. Sizes of molecular mass markers (kDa) are shown on the left.

Using some of the previously described criteria to identify genes with invasion related function (Hinds *et al*., 2009) we identified PF3D7_1017100 (former ID: PF10_0166). According to published studies (Bozdech *et al*., 2003; Le Roch *et al*., 2003) transcription of PF3D7_1017100 increases steadily from 30 h post invasion throughout schizogony. The gene encodes a 310-amino-acid protein of 36.4 kDa. Database searches failed to identify homologues in other Apicomplexan parasites, suggesting that the gene is unique to *Plasmodium* species. Orthologues in other *Plasmodium* species such as *P. vivax* (PVX_001725), *P. knowlesi* (PKH_060120), *P. berghei* (PBANKA_050140) and *P. yoelii* (PY00202) are highly conserved. PBANKA_050140 is most divergent from PF3D7_1017100 but nevertheless shows an overall amino acid sequence identity of 40.2% and a similarity of 59.4% (Fig. 1A). There is an absolute conservation of four cysteine residues in the second half of the protein sequence, suggesting the importance of conservation of higher-order structure between orthologues. The sequence of PF3D7_1017100 in eight different *Plasmodium falciparum* isolates (3D7, Dd2, VS/1, HB3, SenegalV34.04, IT, IGH-CR14, RAJ116) is identical at the nucleotide level (http://www.broadinstitute.org/annotation/genome/plasmodium_falciparum_spp/MultiHome.html). Discordant sequence data for PF3D7_1017100 in D10 and 7H8 lines were checked by DNA sequencing of PCR-amplified PF3D7_1017100 and corrected; both D10 and 7G8 sequence for PF3D7_1017100 are identical to that of 3D7. Based on the location of the protein (see below), the number of proteins described previously with this location and its restriction to the genus *Plasmodium*, we have designated the product of the PF3D7_1017100 gene as RON12.

### RON12 is expressed as a soluble protein in schizonts and merozoites of *P. falciparum*

We amplified the ORF of *Pfron12* without the putative signal peptide sequence and expressed recombinant protein to which we raised both polyclonal rabbit and mouse antibodies. Rabbit antibodies were affinity purified, followed by IgG selection. These antibodies were used on immunoblots of 3D7 schizont, merozoite and uninfected erythrocyte lysates as well as culture supernatant (Fig. 1B) showing a single band at 37 kDa, consistent with the predicted size. After determining that RON12 is expressed late in asexual stages of 3D7 parasites we investigated the solubility profile of this protein. We hypotonically extracted soluble proteins from purified schizonts or merozoites, followed by serial extraction of the insoluble pellet with a high-salt buffer and sodium carbonate buffer, pH 11.0 to separate peripheral from integral membrane proteins. Fractions were separated by SDS-PAGE and immunoblotted (Fig. 1C). In both schizonts and merozoites, RON12 was extracted by hypotonic lysis consistent with a highly soluble protein lacking putative transmembrane regions. Integral membrane proteins used as controls were: MSP2 (GPI-anchored merozoite surface protein) (Gerold *et al*., 1996) and calcium-dependent protein kinase 1 (CDPK1; anchored in plasmalemma by acylation) (Green *et al*., 2008).

### RON12 is located in the neck of rhoptries in merozoites

**Figure 2:**
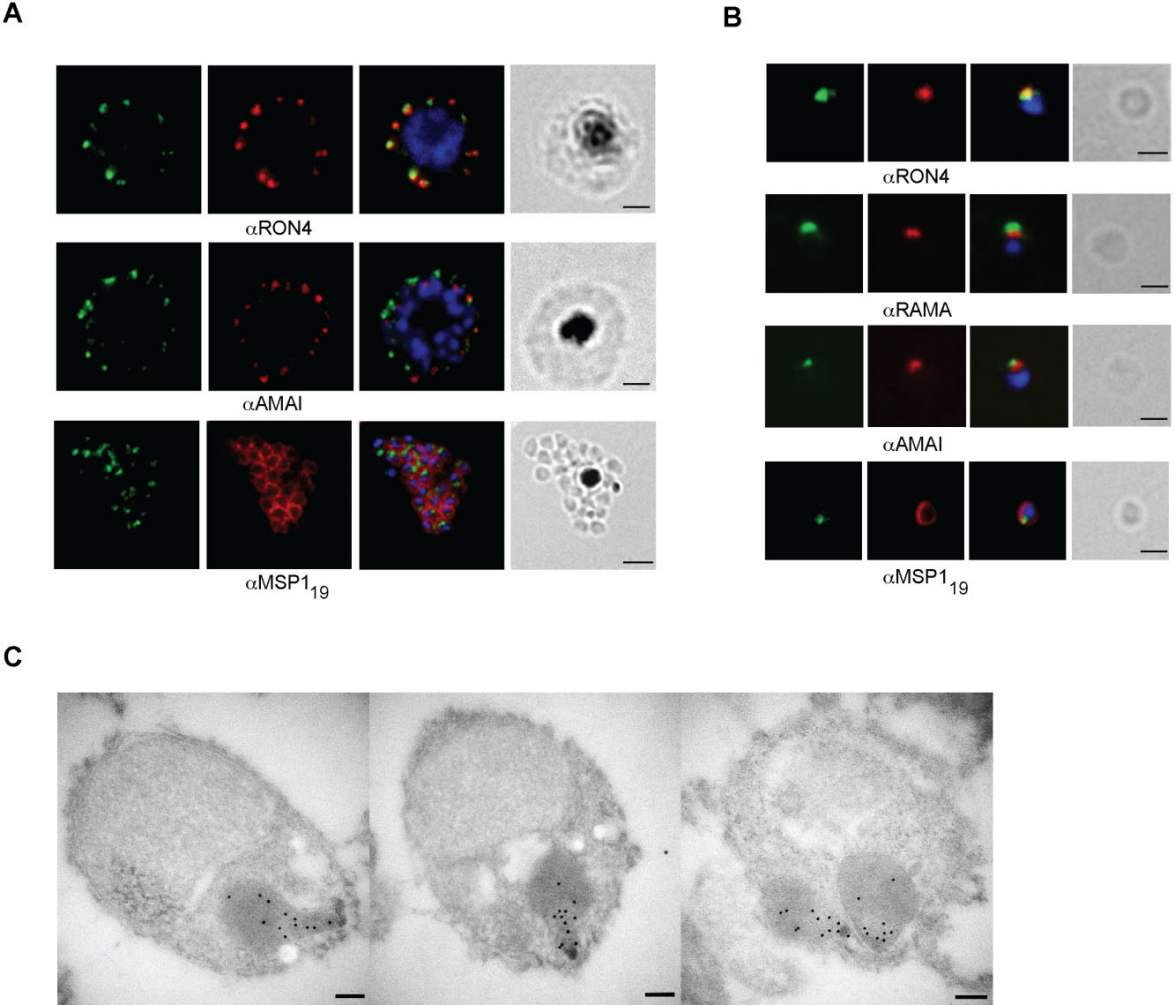
RON12 is located within the rhoptry neck.A and B. Immunofluorescence and bright-field images of schizonts (A) and merozoites (B). Images from left to right show anti-RON12 labelling (green) followed by microneme (anti-AMA1), rhoptry (neck: anti-RON4; body: anti-RAMA) and surface (anti-MSP1_19_) specific antibodies in red, overlay of both with DAPI-stained nuclei and the bright-field image. Size bars in (A) equal 2 μm and in (B) 1 μm.C. Immuno-electron microscopy of isolated merozoites. Localization of RON12 in the rhoptry neck of three different merozoites is shown. Size bars equal 100 nm.

We used the anti-RON12 antibodies to investigate the subcellular location of RON12 by immunofluorescence assay (IFA). In both schizonts and merozoites RON12 appears in a tight apical focus (Fig. 2A and B). By dual labelling with described compartment markers such as AMA1 (micronemes), rhoptry-associated membrane antigen, RAMA (rhoptry body), rhoptry neck protein 4, RON4 (rhoptry neck) and the C-terminal 19 kDa region of merozoite surface protein 1 (MSP1_19_) it appears that RON12 colocalizes with rhoptry markers – apical to the rhoptry bulb and generally coincident with the rhoptry neck marker RON4. No co-staining was seen with either microneme or merozoite surface markers. To further confirm the rhoptry neck location of RON12 we used paraformaldehyde fixed, purified merozoites in immuno-electron microscopy (IEM). In IEM images (Fig. 2C) the RON12-specific antibody labels the apical end of the rhoptries, reminiscent of RON4 labelling (Alexander *et al*., 2006) and clearly distinct from a rhoptry bulb marker such as rhoptry-associated protein 1, RAP1 (Richard *et al*., 2010).

### Location of RON12 during invasion of erythrocytes

**Figure 3:**
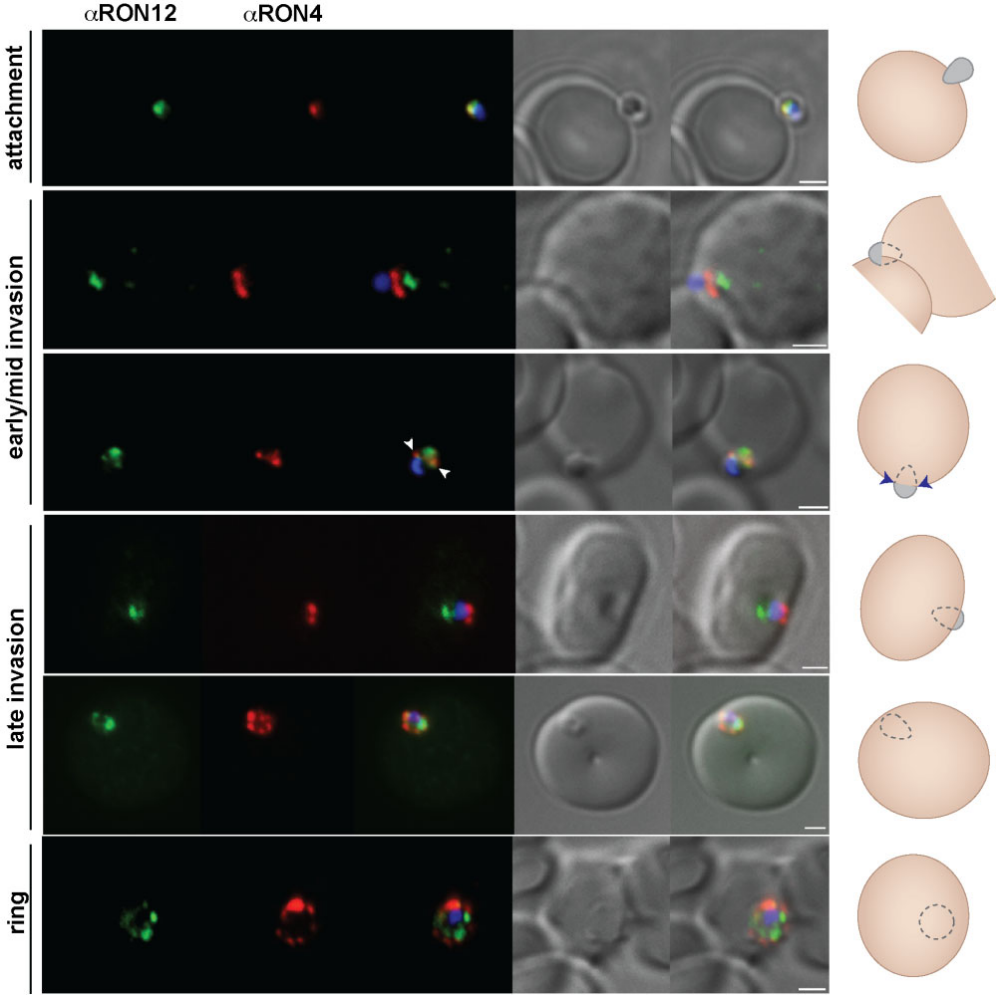
RON12 is predominantly secreted late into the nascent PV. Immunofluorescence and DIC images of merozoites fixed during invasion of erythrocytes. From left to right anti-RON12 (green), anti-RON4 depicting the moving junction (red), overlay of both with nuclear stain DAPI, DIC image and overlay of all images. Cartoon schematic is shown on the right of each panel. Arrowheads depict location of moving junction. Size bars equal 1 μm.

To investigate a possible role of RON12 in host cell entry we visualized RON12 in glutaraldehyde/paraformaldehyde fixed merozoites during erythrocyte invasion. We compared the fluorescent staining pattern of RON12 with that of the validated moving junction marker RON4 (Lebrun *et al*., 2005; Alexander *et al*., 2006), as well as with MSP1, which is shed from the merozoite surface during invasion except for the MSP1_19_ subunit (Blackman *et al*., 1990) and finally with the rhoptry bulb-located RAP1 which is secreted into the nascent PV (Riglar *et al*., 2011). Early in invasion RON12 and RON4 are found together apically located within the rhoptry neck of merozoites attached to erythrocytes (Fig. 3). During early, mid and late stages of invasion, where the apical tip of the merozoite is increasingly protruding into the erythrocyte and the nascent PV being formed, RON4 is part of the MJ, a circumferential band moving from the anterior to posterior of the merozoite and apparent as two points of fluorescence before coming together into the remnant junction of the newly formed ring. However, while examining RON12 and RON4 during invasion we noticed that in the majority of parasites most of RON12 remains apically located within the rhoptry neck of merozoites while the MJ is progressing over the body of the merozoite. Only after the sealing of the erythrocyte plasmalemma is RON12 secreted and then localizes to the PV with a ‘necklace of beads’ appearance. In a total of five invasion events recorded, a proportion of RON12 localizes to the moving junction (Fig. 3, third row of panels with MJ indicated by arrowheads). The retention of a RON in the rhoptry neck during invasion with only a portion of the protein being secreted early and associated with the MJ has previously been reported for another merozoite rhoptry neck protein called apical sushi protein (ASP) (Zuccala *et al*., 2012). It is also not unusual for defined MJ markers to be retained partially in the rhoptry neck throughout invasion as previously described both for RON2 and for RON4 in *T. gondii* tachyzoites (Lamarque *et al*., 2011; Tyler and Boothroyd, 2011). The soluble nature of RON12 as well as its potential weak interaction with MJ components might account for the difficulty in detecting a RON12 association with the MJ. However, we cannot rule out either that the occasional (five events detected) partial association of RON12 with the MJ is not an artefact of fixation. Attempts to immunoprecipitate RON12-interacting proteins such as MJ components have so far been unsuccessful. Comparison of the location of RON12 with that of MSP1_83_ (the N-terminal 83 kDa fragment of merozoite surface protein 1, which is shed during invasion) confirmed that the secretion of RON12 into the PV occurred after completion of invasion, but also appeared to show a potential association with an area on either side of the diminishing MSP1 signal due to shedding, which we interpret to be the MJ (Fig. S1; potential moving junction locations indicated by arrowheads). These images also highlight the fact that the shedding of MSP1 appears to occur at or close to the migrating moving junction. This has long been presumed based on transmission electron microscopy evidence of the lack of a fibrillar surface coat anterior to the moving junction (Aikawa *et al*., 1978) and as previously suggested by comparison of MSP1_83_ with RON4 during invasion (Riglar *et al*., 2011).

**Figure 4:**
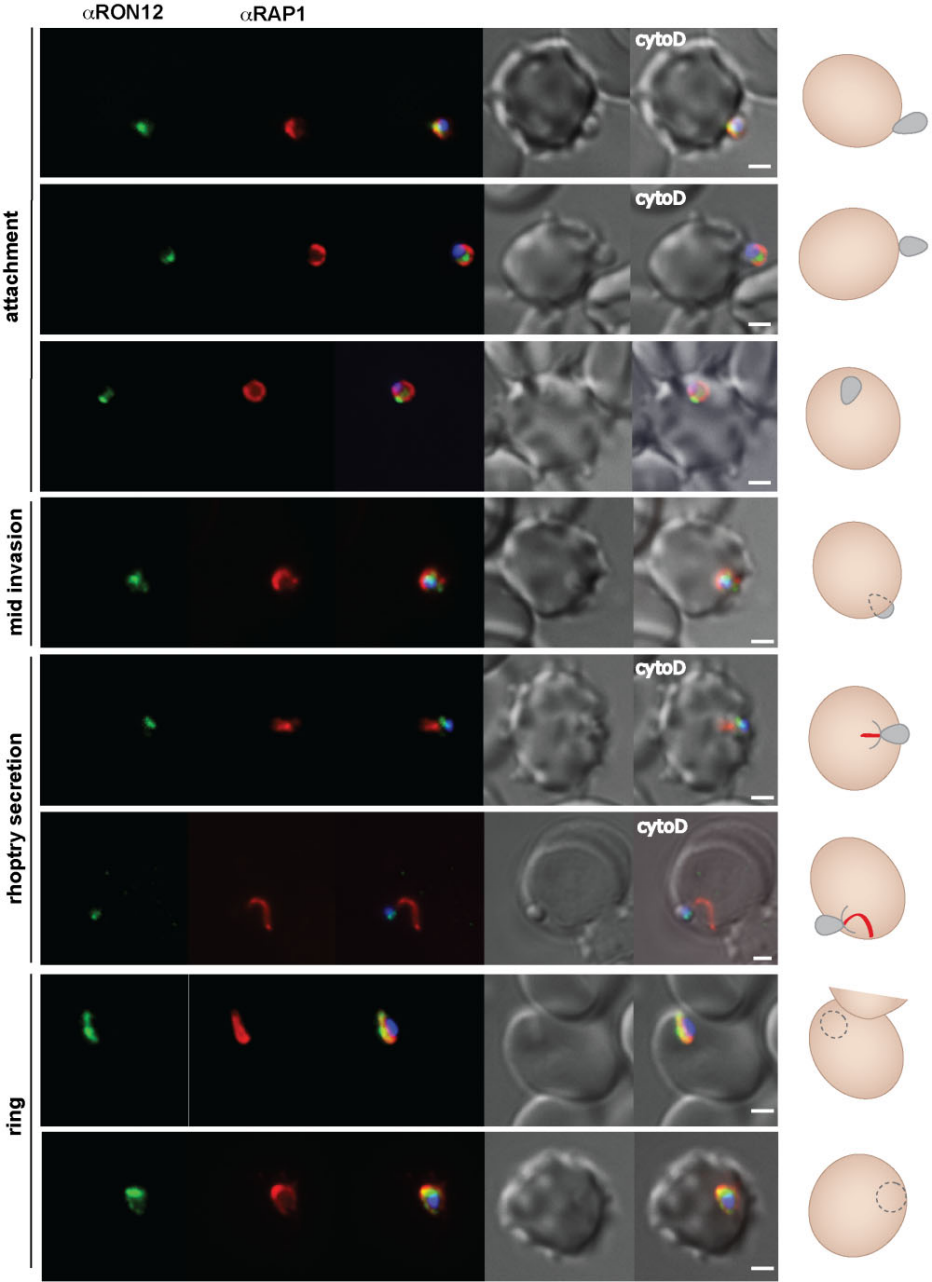
RON12 secretion into the nascent PV during invasion follows that of the rhoptry bulb located RAP1. Immunofluorescence and DIC images of merozoites fixed during invasion of erythrocytes. From left to right: anti-RON12 (green), anti-RAP1 mAb 7H8/50 (red), overlay of both with DAPI nuclear stain, DIC image and overlay of all images. Merozoite attachment in the presence of cytochalasin D (cyto D) is indicated. A cartoon schematic is shown on the right of each panel (red line represents RAP1 secreted into the host cell). Size bars equal 1 μm.

Finally we wanted to investigate the temporal release of RON12 compared with that of the rhoptry bulb marker RAP1 during invasion. Upon contact with the host cell, both in the presence and in the absence of the actin polymerization-inhibiting drug cytochalasin D, invading merozoites showed an apical fluorescence for RON12 whereas RAP1 either was found within the rhoptries or appeared to be released onto the surface of the merozoite. RAP1 released on to the merozoite surface might correspond to aberrant rhoptry bulb release as previously observed in the presence of the inhibitory R1 peptide (Riglar *et al*., 2011). In other examples (Fig. 4, mid-invasion) and during rhoptry expulsion after MJ formation in the presence of cytochalasin D (Fig. 4, rhoptry secretion), RAP1 was evidently released into the nascent PV while RON12 was still associated with the apical tip of the merozoite. Upon completion of invasion both RAP1 and RON12 were present within the newly formed PV of the ring stage. A strong localized staining was detected in these ring stages for both RON12 and RAP1; however, whether or not this marks the remnant junction following completion of invasion needs further investigation (Fig. 4, ring).

These data indicate that RON12 is located in the rhoptry neck, and that its release is delayed compared with that of the rhoptry bulb marker RAP1.

### Real-time imaging of invasion using RON12-transgenic parasites

**Figure 5:**
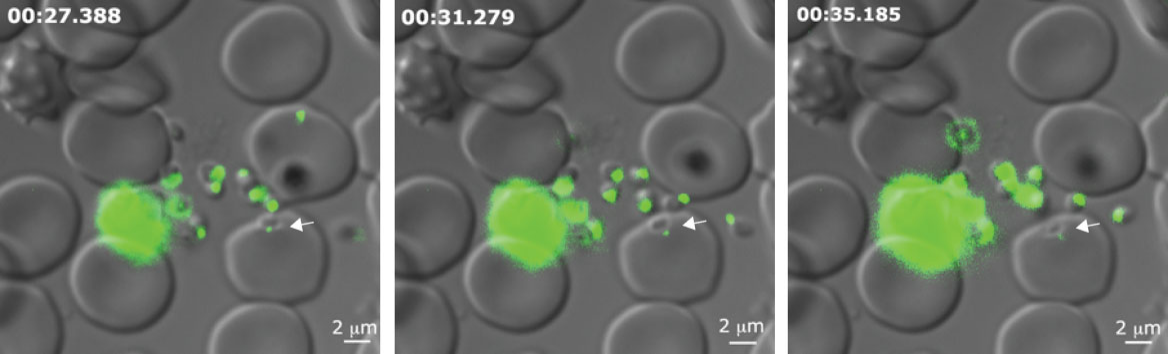
RON12-GFP retains its apical location during erythrocyte invasion, suggesting that secretion into PV occurs late during or after invasion has completed. Images correspond to time points 8, 9 and 10 of invasion Movie S1. Time in min : s.ms corresponds to time each DIC image was taken after schizont rupture. DIC image was taken first followed by GFP image (torrent movement + exposure time = *c*. 2.3 s delay between the corresponding DIC and GFP images). Size bars equal 2 μm.

To further investigate the timing of release of RON12 we generated *ron12-gfp* parasites (Fig. S2). We amplified a 628 bp fragment corresponding to the 3′ end of *Pfron12* ORF and cloned it into the vector pHH3bsdGFP, transfected 3D7 ring-stage parasites, selected with blasticidin-S-HCl and cycled these parasites on and off drug for two cycles before cloning by limiting dilution. To confirm correct integration into the *ron12* locus we analysed genomic DNA by analytical PCR (Fig. S2B) and Southern blot (data not shown) and confirmed the generation of RON12-GFP by immunoblotting with both anti-RON12 and anti-GFP antibodies (Fig. S2C). We verified that the localization to the rhoptry neck was not affected by incorporation of a C-terminal GFP-tag (Fig. S2D). Following generation of *ron12-gfp* parasites we visualized egress and invasion of erythrocytes on an Axioimager M1 fluorescent microscope in real time (Movies S1 and S2). Images were taken approximately every 3.9 s. This is the first time to our knowledge that invasion of *Plasmodium* merozoites was recorded in real-time while simultaneously recording the localization of a GFP-fluorescent invasion molecule. In more than 15 successful invasion events observed we were able to distinguish the previously described three sequential phases of invasion: pre-invasion where the merozoite makes contact with the erythrocyte causing waves of deformation throughout the host cell, a resting phase before commencing penetration of the host cell, and the post invasion echinocytosis phase of the host cell, which was not always detected (Gilson and Crabb, 2009). In Fig. 5 three still images from an invasion series are displayed showing attachment, mid-invasion and late invasion stages of a *ron12-gfp* merozoite. This image series shows the persistence of an apical GFP fluorescence spot throughout invasion, confirming the presence of RON12 in the rhoptry neck until completion of invasion. We were unable to visualize the release of RON12-GFP into the nascent PV by real-time imaging as the GFP fluorescence signal was too weak when released from the rhoptry neck. However, we did detect RON12-GFP in the PV of young ring-stage parasites by IFA.

In summary, we have established that during invasion RON12 remains mainly within the rhoptry neck until the invasion has almost completed before being relocated to the PV. Few examples of a potential association of RON12 with the progressing MJ have also been detected suggestive of a potential role of RON12 within this structure.

### RON12 is a soluble protein located within the PV of ring-stage parasites

Although RON12 was detected in parasite culture supernatant (Fig. 1B), it was also detected by IFA in the nascent PV of newly formed ring stages (Figs 3 and 4, Fig. S1), and therefore we investigated the subcellular location and persistence of RON12 in ring stages following invasion. We biosynthetically labelled mature schizonts and harvested them immediately or allowed merozoites to egress and reinvade fresh erythrocytes. Then samples of equal numbers of parasites were taken corresponding to early (5 h), middle (12 h) or late ring stages (25 h) of development as well as from culture supernatant and used for immunoprecipitation with anti-RON12 and anti-MSP1_19_ antibodies (Fig. S3A). The 37 kDa band corresponding to full-length RON12 persisted throughout the ring stage of the parasite cycle without significant loss over time. RON12 was also detected in the culture supernatant in lesser amount compared with the intracellular RON12. RON12 persists throughout the intracellular cycle in the PV as determined by IFA. The 32 kDa band in the schizont (S) sample is probably an artefactual fragment resulting from non-specific proteolysis induced by detergent treatment. In the extracts of ring-stage parasites, anti-MSP1_19_ antibodies only detected the 19 kDa fragment of MSP1 that is targeted to the food vacuole following invasion (Dluzewski *et al*., 2008), ruling out schizont contamination of either the culture supernatant or the ring-stage samples.

To determine the subcellular location of RON12 in ring-stage parasites we carried out selective permeabilization either of the erythrocyte membrane alone using streptolysin O (SLO) or of the erythrocyte membrane and the PVM using saponin (Ansorge *et al*., 1996) (Fig. S3B). RON12 was solubilized by saponin but not SLO treatment, indicating that RON12 is located in the PV of ring-stage parasites. The ER-marker, BiP, was found in the pellet fraction of both permeabilization conditions, indicating that the parasites remained intact in these procedures.

The data demonstrate that the majority of RON12 from the rhoptry neck is transferred into the PV of ring-stage parasites where it persists throughout the blood-stage cycle as a soluble protein.

### Deletion of *ron12* in human and mouse malaria parasites affects parasite proliferation

**Figure 6:**
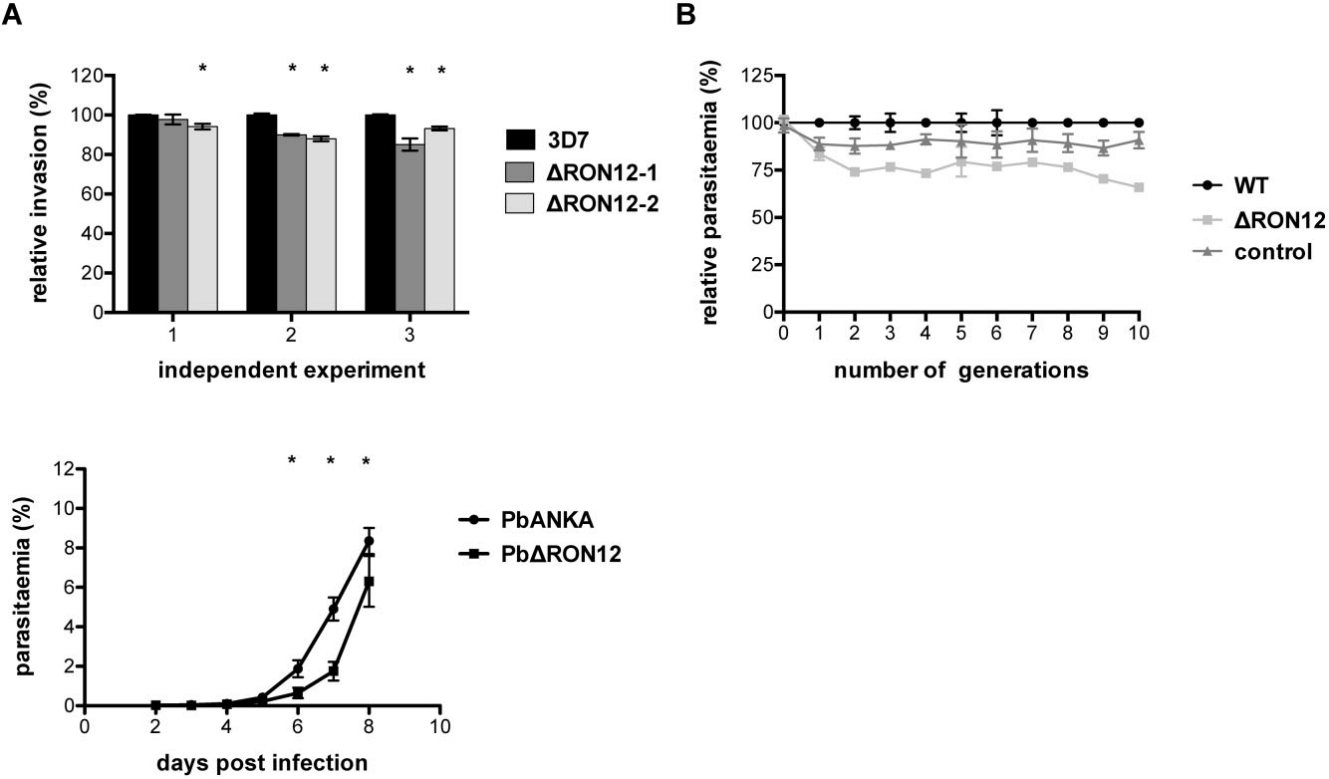
*Pf*Δ*ron12* and *Pb*Δ*ron12* blood-stage parasites show a reduction in parasite numbers compared with wild-type parasites.A. Comparison of relative invasion rate of *Pf*Δ*ron12* and wild-type 3D7 parasites during a single invasion cycle. Three separate experiments using O + blood from three different donors are shown (1, 2, 3). Parasitaemia of triplicate samples was determined by FACS before and after assay set-up and about 32 h following invasion. Means were calculated and converted to invasion rate. Invasion rate of 3D7 was set as 100%. Invasion rate of *Pf*Δ*ron12* clonal lines was calculated and displayed relative to 3D7. Asterisk indicates a significant difference between wild-type and knockout parasites as determined by one-way anova (*P* < 0.05).B. Growth assay of wild-type 3D7 and *Pf*Δ*ron12* over 10 generations. Highly synchronized 3D7 (WT), *Pf*Δ*ron12* and *Pf*Δ*msp3* (control) cultures were diluted to 0.5% parasitaemia in 3% haematocrit. Parasitaemia was measured directly after set-up (parasite generation 0). Subsequently, parasitaemias were determined by FACS counting every 48 h. Cultures were diluted at this point in equal measure. Parasitaemia of wild-type parasites at each point was counted and set at 100%. Parasitaemia of *Pf*Δ*ron12* is displayed relative to wild-type parasite level.C. Growth comparison of wild-type *PbANKA* and *Pb*Δ*ron12* blood-stage parasites *in vivo*. Groups of five BALB/c mice were infected with 1000 blood-stage parasites and the parasitaemia was measured daily by counting a minimum of 3000 cells per Giemsa-stained slide until 8 days post infection. Error bars indicate ± standard deviation. Asterisk indicates significant difference as determined by two-tailed unpaired *t*-test (*P* < 0.05).

We were interested to investigate whether RON12 is essential for survival of blood-stage parasites, as previously described for other rhoptry neck proteins in *Plasmodium* (Proellocks *et al*., 2009; Giovannini *et al*., 2011). For this we generated RON12-knockout parasites by double homologous recombination not only in *P. falciparum* but also in *P. berghei*. For targeting the genomic loci in *P. falciparum* 3D7 and *P. berghei* ANKA we designed transfection constructs in the pHTK (Duraisingh *et al*., 2002) and pBS-DHFR (Dessens *et al*., 1999) vectors respectively (Fig. S4A and D). In both cases the gene was successfully targeted as shown by Southern blot for *P. falciparum* (Fig. S4B) and PCR amplification of the disrupted locus in *P. berghei* (Fig. S4E). The absence of RON12 was confirmed in the transgenic parasites by lack of reactivity with specific antibodies on immunoblots (Fig. S4C and F). The knockout parasites were viable and capable of invading and multiplying within erythrocytes. *Ron12* seems therefore not to be essential for survival of blood-stage parasites under the experimental conditions used. However, close examination of invasion and growth rates in single cycle invasion assays or growth cycle assays over 10 (*P. falciparum*) or 8 generations (*P. berghei*) revealed a small but significant drop in parasitaemia (Fig. 6) for the Δ*ron12* parasites compared with the wild-type parasites in both model systems. For *P. falciparum*, relative invasion rates were determined in three separate experiments comparing wild-type 3D7 to two clonal Δ*ron12* parasite lines over one replication cycle (Fig. 6A). In all three experiments using O + erythrocytes from different donors a small but significant difference was shown for the invasion rates (relative rates ranging from 94% to 85% of wild-type levels) of at least one of the knockout clones compared with 3D7 as determined by one-way anova (*P* < 0.05). When comparing relative parasite growth over 10 generations between 3D7 and *Pf*Δ*ron12* a trend in reduction of growth is evident with *Pf*Δ*ron12* (Fig. 6B). This drop in parasitaemia amounts to 66% compared with that of the wild type after 10 generations but is not always seen from one generation to the next. This reduction in parasitaemia is independent of the hDHFR selection cassette carried in the *Pf*Δ*ron12* parasites as other knockout parasites carrying the same selection cassette in the same growth cycle assay did not show this phenotype (Fig. 6B, control). For the *P. berghei* growth cycle assay the parasitaemia was measured over time in two independent experiments in groups of mice infected with either wild-type or *Pb*Δ*ron12* parasites. Parasitaemia developed more slowly in mice infected with *Pb*Δ*ron12* compared with wild-type *Pb*ANKA showing a delay of approximately 1 day before reaching similar parasitaemias as the wild type with the curves otherwise showing a highly similar profile (Fig. 6C). The differences in parasitaemias were significant on days 6, 7 and 8, after which the experiment was terminated. Similar growth defects seen in the asexual replication cycles have been reported previously for knockout studies involving both proteases and kinases of *P. berghei* (Spaccapelo *et al*., 2010; Tewari *et al*., 2010). These knockout parasites were like *Pb*Δ*ron12* able to reach similar parasitaemias as wild-type parasites but took longer to reach these. These results from independent experiments using different parasite species, indicate that although RON12 is not essential for asexual parasite growth under the conditions used, its absence imparts a detectable disadvantage in these knockout parasites affecting parasite numbers. It is unclear whether this is a direct effect due to reduced invasion or whether reduced parasite growth contributes to the reduction in parasitaemia. We can however rule out differences in multiplication rates due to differences in merozoite numbers adversely affecting parasite numbers and also can rule out significantly different cell cycle timings as the cause for the reduced parasitaemias detected in the knockout parasites. Phenotypical analyses using wild-type 3D7 and *Pf*Δ*ron12* parasites did not identify clear differences in merozoite numbers per schizont averaging between 20 and 21 merozoites per schizont (Fig. S5A). Determination of the timing of the asexual growth cycle showed a 1–1.5 h delay in reaching the same ratio of released parasites to schizonts in the *ron*12 knockout lines (Fig. S5B). However, this difference lies within the experimental set-up window of 1 h and therefore is not significant. Equally a control parasite line having the *msp3* ORF disrupted by the same *hDHFR* cassette used in generating *Pf*Δ*ron12* parasites showed a slightly prolonged cycle time (1.5–2 h) compared with wild-type parasites but showed no significant change in parasitaemia after 10 generations (Fig. 6B).

## Discussion

A number of rhoptry neck proteins have recently been described. Some are involved in MJ formation such as RON1 (ASP), 2, 4, 5 and TgRON8 (Lebrun *et al*., 2005; Straub *et al*., 2009; Zuccala *et al*., 2012), whereas others have not been found to participate in the MJ, namely RON3, TgRON9, TgRON10 and TgRON11 (Ito *et al*., 2011; Lamarque *et al*., 2012; Beck *et al*., 2013). Some have not been studied in sufficient detail to determine their localization during invasion, such as RON6 and Pf34 (Proellocks *et al*., 2007; 2009). Of the MJ RONs RON2 and 4 have been shown or are believed to be essential, whereas TgRON8 has a critical anchoring function of the MJ to the host cell cytoskeleton but is not essential under the experimental conditions used (Giovannini *et al*., 2011; Srinivasan *et al*., 2011; Straub *et al*., 2011). RON1, 2, 3, 4, 5, 6 and RON11 are found throughout the phylum of Apicomplexa whereas RON8, 9 and 10 are coccidia specific.

Here we have described a novel rhoptry neck protein, RON12, which unlike all other previously characterized RONs is restricted to *Plasmodium* and constitutes a completely soluble protein devoid of any tight membrane associations. A previous study reported an apical localization for an episomally expressed copy of RON12 fused to GFP and suggested a microneme location (Hu *et al*., 2010). We have shown here unequivocally a location in the rhoptry neck for the endogenous protein, both by IFA colocalization studies with RON4 and also by IEM. Like some other RONs, RON12 appears to be able to associate at least in part with the MJ during erythrocyte invasion by merozoites. This association appears to be weak and was therefore only detected in a minority of invasion events requiring further confirmation using different experimental approaches. The majority of RON12 however is secreted into the nascent PV after completion of invasion, similar to a recently described *Plasmodium* protein, PFF0645c, which is the homologue of TgROP14 and is located in the rhoptry membrane or at the posterior of the rhoptry bulb in membranous structures (Zuccala *et al*., 2012). This is quite remarkable since RON12 remains localized at the exit point of the rhoptries during protein secretion from the bulb and posterior end of the rhoptries. This points to some compartmentalization within the narrow rhoptry neck allowing the release of bulb components in membranous whorls for example, through the centre of the channel. This observation does not concur with the recently proposed timing of protein release from rhoptries based on rhoptry architecture (Zuccala *et al*., 2012). To address the timing of release of RON12 in real time we generated a parasite line in which we fused GFP to the C-terminus of the endogenous *Pfron12* ORF allowing us to follow RON12-GFP during invasion. We did not detect the association of RON12-GFP with the moving junction, either because too little protein associates with the MJ to be visualized by epifluorescence microscopy or because the C-terminal GFP fusion interfered with this association. We did however record the late release of RON12-GFP following successful invasion (as disappearance of the rhoptry neck GFP signal). RON12-GFP parasites generated by single site homologous recombination were not stable in culture. Even under continued drug pressure these parasites reverted to wild type having lost the C-terminal GFP fusion. This seems to suggest that GFP at the C-terminus of RON12 interferes with its function and maybe compromises its participation in the MJ or affects its role within the PV of ring-stage parasites.

A comparable bipartite secretion from the rhoptries, has been observed recently for an epitope-tagged version of RON1 (ASP), a GPI-anchored rhoptry neck protein (Zuccala *et al*., 2012). In a transgenic parasite line in with a HA tag was fused to the C-terminus of ASP, which likely resulted in an altered membrane attachment (transmembrane versus GPI), ASP-HA showed both an association with the MJ and a late-stage secretion from the rhoptry neck. The localization of ASP following invasion has not been addressed. It should be noted that even bona fide MJ markers such as RON2, RON4 and TgRON8 are retained to some extent within the rhoptry neck during invasion of tachyzoites while a portion of the protein is associated with the progressing MJ (Straub *et al*., 2009; Lamarque *et al*., 2011). It is currently unclear what happens to the protein retained within the rhoptry neck. Following successful invasion RON12 can be found in ring-stage parasites as a soluble protein within the PV and persisting throughout the asexual life cycle. This is unlike other characterized MJ components which localize to the host cell side of the PVM at the commencement of invasion, namely RON4, 5 and TgRON8 (Besteiro *et al*., 2009), and anchor the moving junction to the underlying host cell cytoskeleton. Whether any of these characterized junction proteins have a function following successful invasion is currently unknown.

Peptides corresponding to RON12 have been identified in proteomes of blood-stage schizonts and merozoites as well as sporozoites (Lasonder *et al*., 2008; Treeck *et al*., 2011), suggesting a *Plasmodium*-specific invasion function irrespective of host cell type (erythrocyte and hepatocyte). Rhoptry proteins involved in the MJ (RON1, 2 and 5) and potential PVM formation (RhopH 3 and RAP2) have also been detected in the sporozoite proteome but rhoptry neck tip located RH proteins which are involved in receptor recognition (Tham *et al*., 2012) have not, suggesting that RON12 is not involved in host cell receptor binding. Unsurprisingly, no RON12 was identified in the proteome of ookinetes, as these motile stages lack rhoptries (Patra *et al*., 2008).

As with other rhoptry proteins, the amino acid sequence of RON12 is highly conserved between different *P. falciparum* isolates, which might be a consequence of inaccessibility to functional antibodies and therefore the lack of selection pressure to avoid immune detection, or it might reflect the importance of the protein's structure for its function. Phenotype analysis of RON12-knockout parasites in both *P. falciparum* and *P. berghei* confirms the importance of RON12 during the blood-stage cycle of *Plasmodium*. *ron12*-knockout parasites in both species show growth retardation compared with wild-type parasites. It is currently unclear whether the lack of RON12 has a negative effect on invasion or whether its absence in the PV of ring-stage parasites causes parasite death during development as merozoite numbers per schizont seem comparable between wild-type and knockout parasite lines as are the timings for asexual cycle length.

Given the localization data for RON12 in merozoites during invasion and in newly formed ring-stage parasites, we suggest that one role of RON12 might involve steps in the establishment of the PV of the intracellular parasite, such as involvement in protein export or nutrient acquisition and this could explain its continued persistence in this location throughout the cycle. RON12's presence in the culture supernatant and its potential weak association with the MJ suggest that this protein might also be able to be involved with the parasite site of the MJ; however, further studies are required to establish potential interaction partners.

## Experimental procedure

### Ethics statement

All animal work protocols were reviewed and approved by local Ethical Review and approved and licensed by the UK Home Office as governed by law under the Animals Scientific Procedures Act 1986 (Project licences 40/3344 and 70/7051) and in compliance with ‘European Directive 86/609/EEC’ for the protection of animals used for experimental purposes. Antibodies were purchased from Harlan Laboratories, UK.

### Parasite culture and transfection

*Plasmodium falciparum* parasites (3D7 strain) were grown *in vitro* in RPMI 1640 medium containing Albumax II as described previously (Trager and Jensen, 1976) and transfected using standard protocols (Duraisingh *et al*., 2002). Transfected lines were selected with 10 nM WR99210 (kind gift of Jacobus Pharmaceuticals), 10 μM ganciclovir (Sigma Aldrich) or 2.5 μg ml^−1^ blasticidin-S-HCl (Merck Millipore) and cloned by limiting dilution.

*Plasmodium berghei* ANKA parasites were transfected with KpnI/SacII/PvuI-linearized transfection constructs as described (Janse *et al*., 2006). Generation of transfection constructs is described in supporting experimental procedures.

### Parasite phenotypic analysis

To analyse the effect of the lack of PfRON12 in the transgenic parasites on their capability of host cell invasion and growth compared with wild-type parasites, we conducted invasion assays over one multiplication cycle as well as over 10 generations. Parasite cultures with 3% haematocrit and a starting parasitaemia of 0.5% synchronized trophozoite population were set up using O+ erythrocytes. Parasitaemia after set-up and again after 48 h was determined by FACS analysis as described previously (Bergmann-Leitner *et al*., 2006). In experiments over a single invasion cycle the invasion rate for each parasite line cultured in triplicate was determined by dividing the final by the starting parasitaemia. In growth cycle experiments lasting over 10 generations, the starting parasitaemias of duplicate 5 ml cultures of wild type and two clonal *Pf*Δ*on12* lines and two clonal control lines containing the hDHFR cassette in the *msp3* ORF were determined at cycle 0, followed by parasitaemia measurement every 48 h by FACS. Parasite cultures were diluted every 48 h in equal measure into fresh tissue culture plates.

For phenotype analysis *in vivo* we compared parasitaemias of groups of five BALB/c mice injected with 1000 wild-type *P. berghei* ANKA or *Pb*Δ*ron12* parasites over 8 days. Three thousand erythrocytes were counted per Giemsa-stained slide and the parasitaemias recorded. Means were calculated from parasitaemias of four mice from each group.

### Protein expression, antisera production and immunoblot analysis

Open reading frames (ORF) of PF3D7_1017100 and PY00202 lacking the putative signal peptide sequence were amplified for cloning into expression vectors pET-46Ek/LIC and pET-32Xa/LIC (Merck Millipore) respectively. The template for amplification of PF3D7_1017100 was a codon-optimized gene (mammalian codon bias; Geneart), and *P. yoelii* genomic DNA was used for PY00202. Recombinant proteins were produced in *Escherichia coli BL21(DE3)pLysS* cells (rPfRON12) or *E. coli BL21(DE3)* cells (rPYRON12) and purified using non-denaturing conditions. Purified recombinant proteins were used for antibody production in rabbits (Harlan Laboratories) and mice following standard procedures. Rabbit antibodies were subsequently affinity-purified using rPfRON12 coupled to CNBr-activated Sepharose 4B (GE Healthcare) and IgG-selected using protein G-Sepharose (Sigma Aldrich). For Western blot analysis purified parasites were lysed in SDS sample buffer and boiled at 95°C for 5 min. Lysate corresponding to approximately 2 × 10^6^ schizonts or 2 × 10^7^ merozoites was separated on pre-cast 12% Bis Tris NuPAGE polyacrylamide gels (Invitrogen) and transferred to nitrocellulose membranes by electroblotting. Membranes were blocked, and incubated with rabbit anti-PfRON12 antibody (1:8000), mouse anti-MSP2 (1:1000; Gerold *et al*., 1996), rabbit anti-CDPK1 (1:1000; Green *et al*., 2008); rat anti-BiP (1:1000), mouse anti-GFP (1:1000, Roche) and mouse anti-PYRON12 (1:1000). Bound antibodies were detected with horseradish peroxidase-conjugated secondary antibodies (Bio-Rad).

### Differential protein extraction and subcellular fractionation

A method adapted from Papakrivos *et al*. (2005) was used. We solubilized purified schizonts and merozoites first in 20 pellet volumes of hypotonic lysis buffer (10 mM Tris, pH 8.0, 5 mM EDTA) followed by centrifugation at 100 000 *g* for 30 min at 4°C. The pellet was washed once in the hypotonic lysis buffer and subsequently extracted with high-salt buffer (10 mM Tris, pH 7.5, 500 mM NaCl, 5 mM EDTA), washed again and then finally extracted in carbonate buffer (100 mM sodium carbonate, pH 11.0). All buffers and incubations were carried out at 4°C and contained complete protease inhibitor cocktail (Roche). SDS sample buffer was mixed with each supernatant after centrifugation and the final carbonate pellet. To investigate the location of PfRON12 within RBC containing ring-stage parasites we extracted them with either streptolysin O (SLO; Sigma Aldrich) or 0.15% saponin as described previously (Rug *et al*., 2004), followed by SDS-PAGE separation and Western blotting.

### Indirect immunofluorescence and IEM assays

Indirect IFA on purified schizonts and released merozoites was performed on air dried thin smears of parasites on glass slides which were fixed in 4% paraformaldehyde for 30 min followed by permeabilization for 10 min in 0.1% Triton X-100 in PBS before blocking in 3% BSA in PBS overnight at 4°C. All antibodies were diluted in 3% BSA in PBS and all incubations lasted 1 h at room temperature (RT). Primary antibodies used were rabbit anti-PfRON12 (1:6000), mouse anti-PfRON12 (1:2000), mAb 24C6 anti-RON4 (1:1000; Roger *et al*., 1988), mouse anti-AMA1 (1:250), mAb 1E1 anti-MSP1_19_ (1:1000), rabbit anti-RAMA (1:500; Topolska *et al*., 2004), rabbit anti-GFP (1:1000; E. Knuepfer and A.A. Holder, unpublished) and mouse anti-GFP (1:500, Roche). After incubation with primary antibodies slides were washed in PBS for 30 min, before incubation with secondary AlexaFluor-labelled (488 and 594; Invitrogen) antibodies at 1:5000. Nuclei were stained with DAPI (4′,6-diamidino-2-phenylindole). Slides were mounted in Vectashield and viewed on a Zeiss Axioplan 2 imaging system with Plan Apochromat 100×/1.4 oil immersion objective. Images were captured using Axiovision 4.6.3 software and edited using Adobe Photoshop.

IFAs of merozoites during the invasion process were performed as described in Riglar *et al*. (2011). In short, tightly synchronized 3D7 schizonts were purified and incubated in medium containing 10 μM E64 (Sigma Aldrich) until maturity, E64 was then washed out and schizonts were incubated with erythrocytes at 37°C under vigorous shaking (5% haematocrit) or filtered through a 1.2 μm acrodisc syringe filter (Satorius) as described previously (Boyle *et al*., 2010). To stop active erythrocyte invasion 1 μM cytochalasin D (Sigma Aldrich) was included in the medium. Samples were taken after 2, 5, 10 min and 30 min (+ cytochalasin D) and directly fixed in solution at a final concentration of 4% paraformaldehyde/0.01% glutaraldehyde for 60 min at RT. Cells were collected by centrifugation at 1800 *g* for 3 min and permeabilized with 0.1% Triton X-100 in PBS for 10 min before blocking in 3% BSA in PBS overnight. Primary and secondary antibody incubations were carried out with the cells in suspension. Affinity-purified anti-PfRON12 rabbit antibodies (1:5000) were used for 1.5 h at RT, followed by three washes of PBS, addition of secondary anti-rabbit AlexaFluor 488 antibody (Invitrogen) diluted in 3% BSA in PBS at 1:4000 for 1.5 h. After three PBS washes anti-RON4 mAb 24C6 (1:500), anti-MSP1_83_ mAb 89.1 (1:2000; Holder and Freeman, 1982) or anti-RAP1 mAb 7H8/50 (1:50; Schofield *et al*., 1986) were incubated for 1.5 h before PBS washes and incubation with AlexaFluor 594 secondary antibodies (Invitrogen) and 0.2 μg ml^−1^ of DAPI for 1.5 h. After a further three washes in PBS, cells were settled onto polyethyleneimine coated slides and sealed with a coverslip. Slides were viewed on a Zeiss Axioimager M1 imaging system with Plan Apochromat 100×/1.4 oil immersion objective. Images were captured using Axiovision 4.6.3 software and edited using Adobe Photoshop.

For IEM purified 3D7 merozoites were fixed in either 4% paraformaldehyde or 2% paraformaldehyde and 0.075% double distilled glutaraldehyde in RPMI 1640 medium for 20 min at 4°C. Samples were then processed for LR White resin embedding (Agar Scientific) and polymerized at RT using indirect ultraviolet light. Thin sections were mounted on nickel grids and immunostained using affinity-purified rabbit anti-PfRON12 antibodies at 1:100. Antibody labelling was detected with protein A-conjugated to 10 nm gold (a kind gift from Dr Pauline Bennett, Kings College London) and examined on a Hitachi 7600 microscope. Control samples were incubated with a polyclonal rabbit anti-MSP1_19_ antiserum (Dluzewski *et al*., 2008).

### Live cell imaging

Tightly synchronized schizonts were collected by centrifugation on 70% Percoll and put back into culture with RPMI 1640-Albumax II and merozoite release was monitored by examining Giemsa-stained smears every 10 min. Parasites were then collected by centrifugation and mixed with erythrocytes in RPMI Albumax II to a final schizont parasitaemia of 20% and a 2% haematocrit. Two microlitres of culture was transferred to glass slides covered with a coverslip whose edges were dipped into Vaseline to generate a shallow incubation chamber. Slides were viewed immediately on the Zeiss Axioimager M1 imaging system with heated stage and Plan Apochromat 100×/1.4 oil immersion objective. Images were captured and processed using Axiovision 4.6.3 software. Real-time image acquisition was programmed to first capture the DIC image followed by the GFP epifluorescence image. Time delay caused by exposure time plus filter torrent movement accounts for about 2.3 s between DIC and GFP images. On composite images (and movies) the time indicated in min : s.ms is the time the DIC image was taken after merozoite egress. To compensate for photobleaching of GFP due to repeated excitation we manually adjusted brightness and contrast settings of the GFP fluorescence channel. Following frame 10, gamma settings of GFP fluorescence signal intensity were altered to aid clarity of rhoptry neck localization. Movies were generated with a frame rate of 5 per second using Axiovision 4.6.3.

## Supporting Information

Supplementary Experimental Methods.

**Fig. S1.** RON12 transfers to the nascent PV of ring stages whereas MSP1_83_ is shed during invasion. Immunofluorescence and DIC images of merozoites fixed during invasion of erythrocytes. From left to right: anti-RON12 (green), anti-MSP1_83_ (mAb 89.1) in red, overlay of both with nuclear stain DAPI, DIC image and overlay of all images. A cartoon schematic is shown on the right of each panel. Arrowheads depict location of a putative MJ. Size bars equal 1 μm.

**Fig. S2.** Generation of transgenic Pfron12-gfp parasites. A. Schematic presentation of GFP-tagging strategy for the endogenous *pfron12* gene locus using the pHH1-derived pHH3ron12-gfp vector. B. PCR analysis of integration of pHH3ron12-gfp into *pfron12*. After two cycles on and off drug selection parasites were cloned by limiting dilution. Amplification with for/rev primers produced a 954 bp product from the endogenous and transgenic locus. Amplification with for/rev2 primer pair amplified 1662 bp product only from transgenic parasite clones showing successful integration. C. Immunoblot of total parasite lysate from wild-type 3D7 or RON12GFP clones 1 and 2 probed with anti-RON12 and anti-GFP antibodies. Sizes of molecular mass markers (kDa) are shown on the left. D. Immunofluorescence analysis of merozoites (top row) and schizonts (bottom row). Parasites were labelled with anti-GFP in green and anti-RON12 or anti-RON4 in red as indicated. Third panel shows an overlay of all the images with the nuclear marker DAPI. Size bars equal 1 μm for merozoite and 2 μm for schizont images.

**Fig. S3.** RON12 is secreted into the PV of developing parasites and persists during intracellular parasite development. A. Schizonts were metabolically labelled for 30 min then either harvested immediately or purified and allowed to develop in unlabelled medium into ring/early trophozoite stages. Immunoprecipitation with either anti-RON12 antibody or anti-MSP1_19_ antibody was carried out from each time point sample: S (schizonts), CS (culture supernatant), 5 (5 h rings), 12 (12 h rings), 25 (25 h late ring/trophozoites). Immune complexes were separated by SDS-PAGE under reducing conditions and visualized by autoradiography. B. Solubility analysis of RON12 in ring-stage parasites. Ring infected erythrocytes (0–3 h old) were treated with streptolysin O (SLO) or saponin. Supernatant (S) and pellet fractions (P) were separated by SDS-PAGE and immunoblotted. Detection with anti-RON12 or anti-BiP antibodies is shown. Sizes of molecular mass markers (kDa) are shown on the left of the panels.

**Fig. S4.** Disruption of *ron12* in *P. falciparum* and *P. berghei*. A. Schematic of double homologous recombination of pHTKron12 into the endogenous locus resulting in the disruption of *Pfron12*. B. Southern blot of genomic wild-type and transgenic parasite DNA (wt, 3D7; 0, cycle 0; 0G, cycle 0 ganciclovir-resistant; 3, cycle 3; 3G, cycle 3 ganciclovir-resistant; clones 1 and 2 generated by limiting dilution) digested with HpaI, separated on a 0.8% agarose gel in TAE buffer and probed with F1 flank of *Pfron12*. Molecular size markers are indicated on left (in kb). C. Immunoblot of total schizont lysate from 3D7 wild-type and Δron12 parasites probed with anti-RON12 and anti-BiP antibodies. Sizes of molecular mass markers (kDa) are shown on the left of the panels. D. Schematic for double homologous recombination of linearized pBS-DHFR-PbRON12 into *Pbron12* locus causing disruption of the locus by integration of the selectable marker TgDHFR-TS. E. PCR analysis for integration. Using primer pair c/d as indicated in (D) results in an 873 bp band in the case of integration whereas using primer pair a/b will amplify the *Pbron12* ORF only when wild-type locus is present (594 bp product). Wt, PbANKA gDNA; 3, third passage under pyrimethamine selection; Δ*PbRON12* clone by limiting dilution from third passage blood. F. Immunoblot of total lysate from *P. berghei* wild-type and Δ*PbRON12* schizonts. Immunoblot was probed with anti-PyRON12 antiserum and anti-BiP as loading control. Sizes of molecular mass markers (kDa) are shown on the left of the panels.

**Fig. S5.** Analysis of multiplication rate and cell cycle length of *Pf*Δ*ron12* parasites. A. Multiplication rate analysis of wt and two *Pf*Δ*ron12* parasites clones. Merozoite numbers within E64-treated mature schizonts were counted on Giemsa-stained slides. Displayed is the mean of means from three independent experiments. Error bars indicate ± standard deviation. B. Determination of cell cycle length for wt, two *Pf*Δ*ron12* parasites clones and a control parasite containing the *hDHFR* cassette integrated into the *msp3* ORF. The ratio of egressed parasites (rings + merozoites) to schizonts was determined by counting Giemsa-stained smears every 30 min starting 39 h after tightly synchronizing the parasites to within a 1 h window (see supplementary Material and Methods). Triplicate samples were counted. Error bars indicate ± standard deviation.

**Fig. S6.** Plasmid map of vector pHH3bsdGFP. pHH3bsdGFP plasmid is displayed with *GFPmut2* ORF in green, *hrp2* 3'utr in dark grey, *Pfhsp86* promoter in blue, *BSD* ORF in yellow and *PbDT* 3'utr in light grey. Restriction enzyme sites shown correspond to unique sites designed for cloning purposes. Total plasmid size is 6117 bp.

**Movies S1 and S2.** Live cell imaging of PfRON12-GFP merozoite invasion into human erythrocytes. Two invasion events are shown in independent movies. Time in min : s.ms corresponds to time when each DIC image was taken relative to schizont rupture. DIC image was taken followed by the GFP image (torrent movement + exposure time = approximately 2.3 s delay between each corresponding DIC and GFP image). Size bars equal 2 μm.
