# Supplementary figures and images for: RON12, a novel Plasmodium-specific rhoptry neck protein important for parasite proliferation
Source: Cell Microbiol. 2013 Aug 28;16(5):657–72. doi: 10.1111/cmi.12181 (PMC3922828; doi:10.1111/cmi.12181)

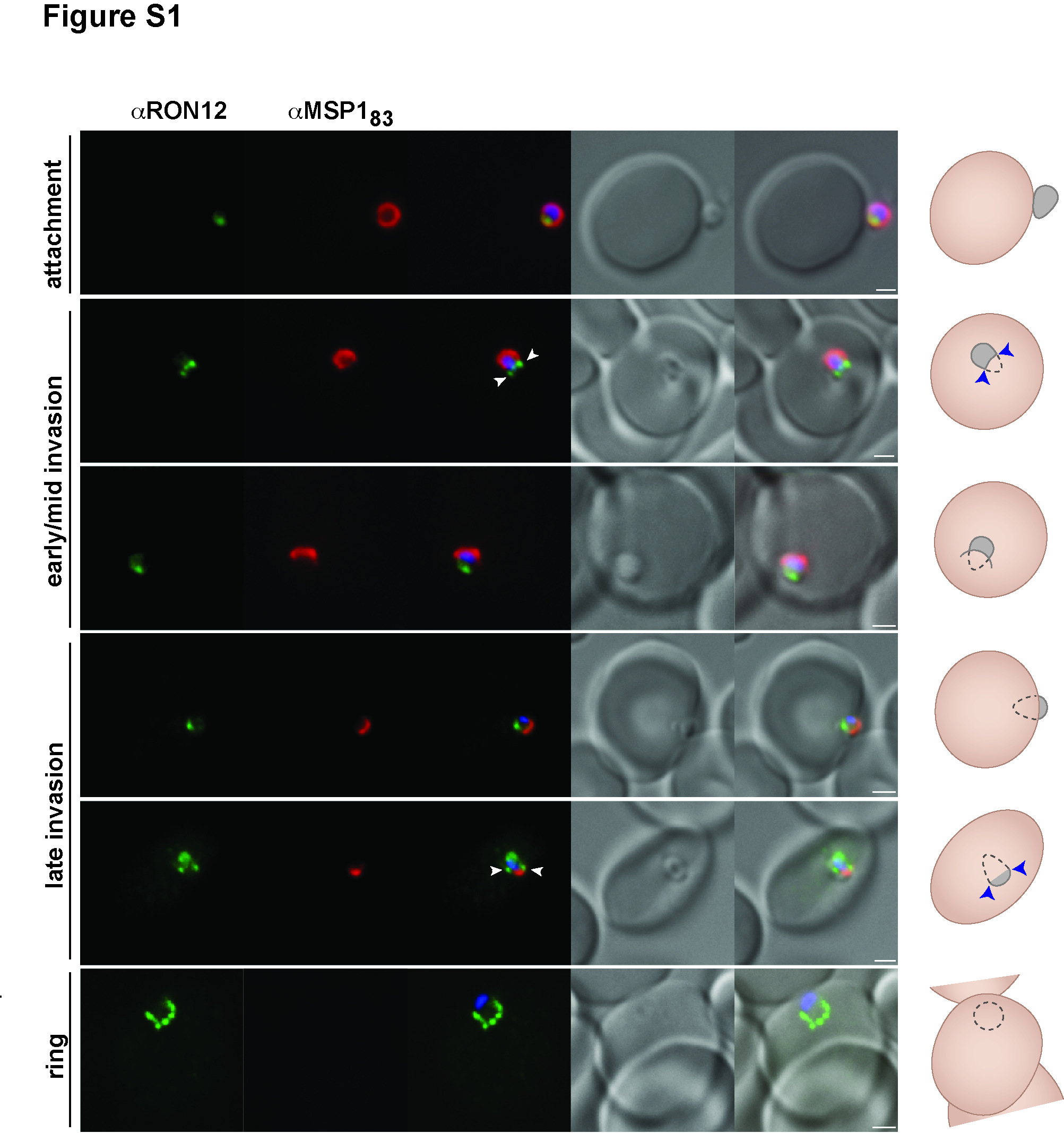

Supplement: Supplementary file 1 [file cmi0016-0657-SD1.tif]

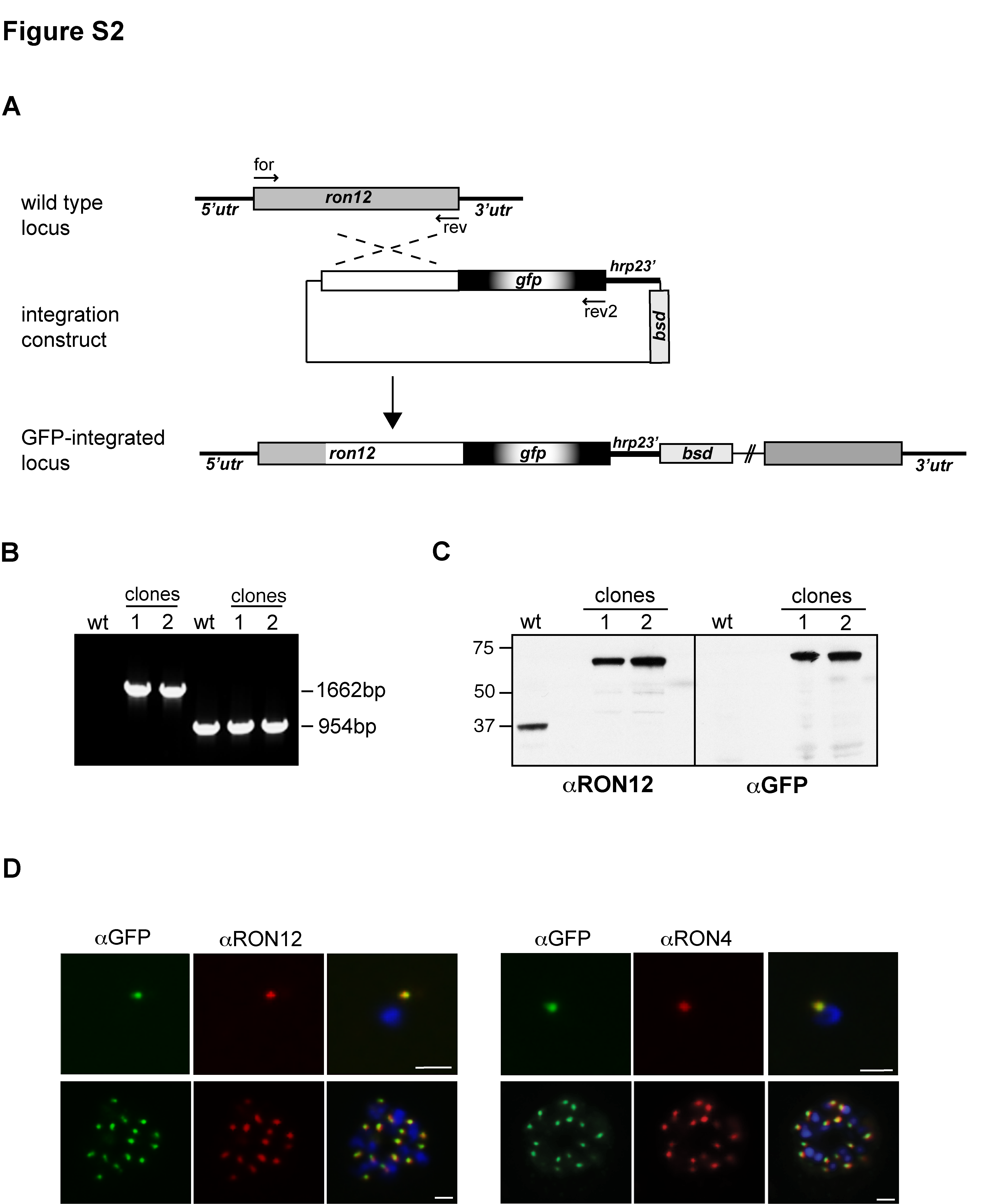

Supplement: Supplementary file 2 [file cmi0016-0657-SD2.tif]

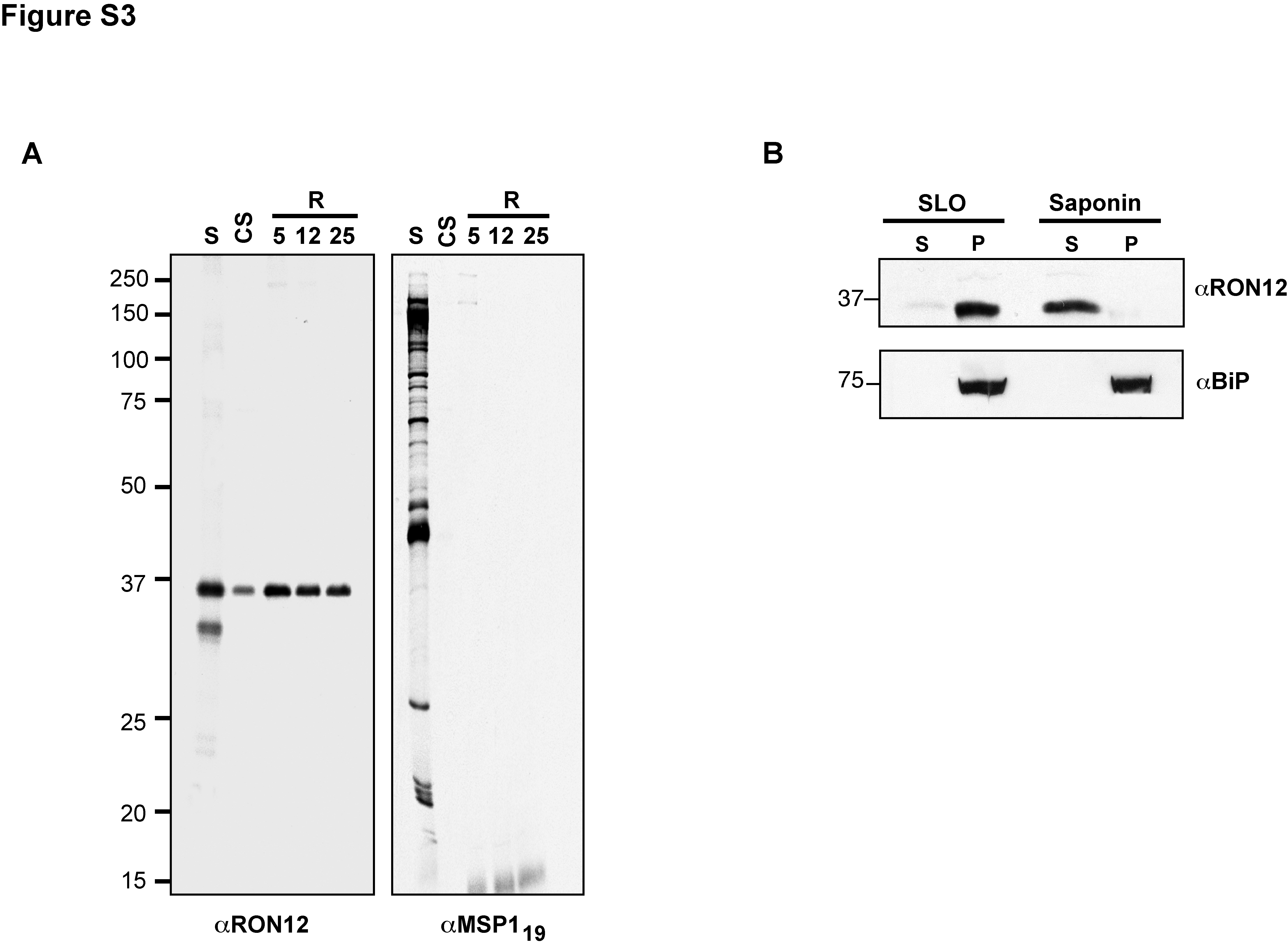

Supplement: Supplementary file 3 [file cmi0016-0657-SD3.tif]

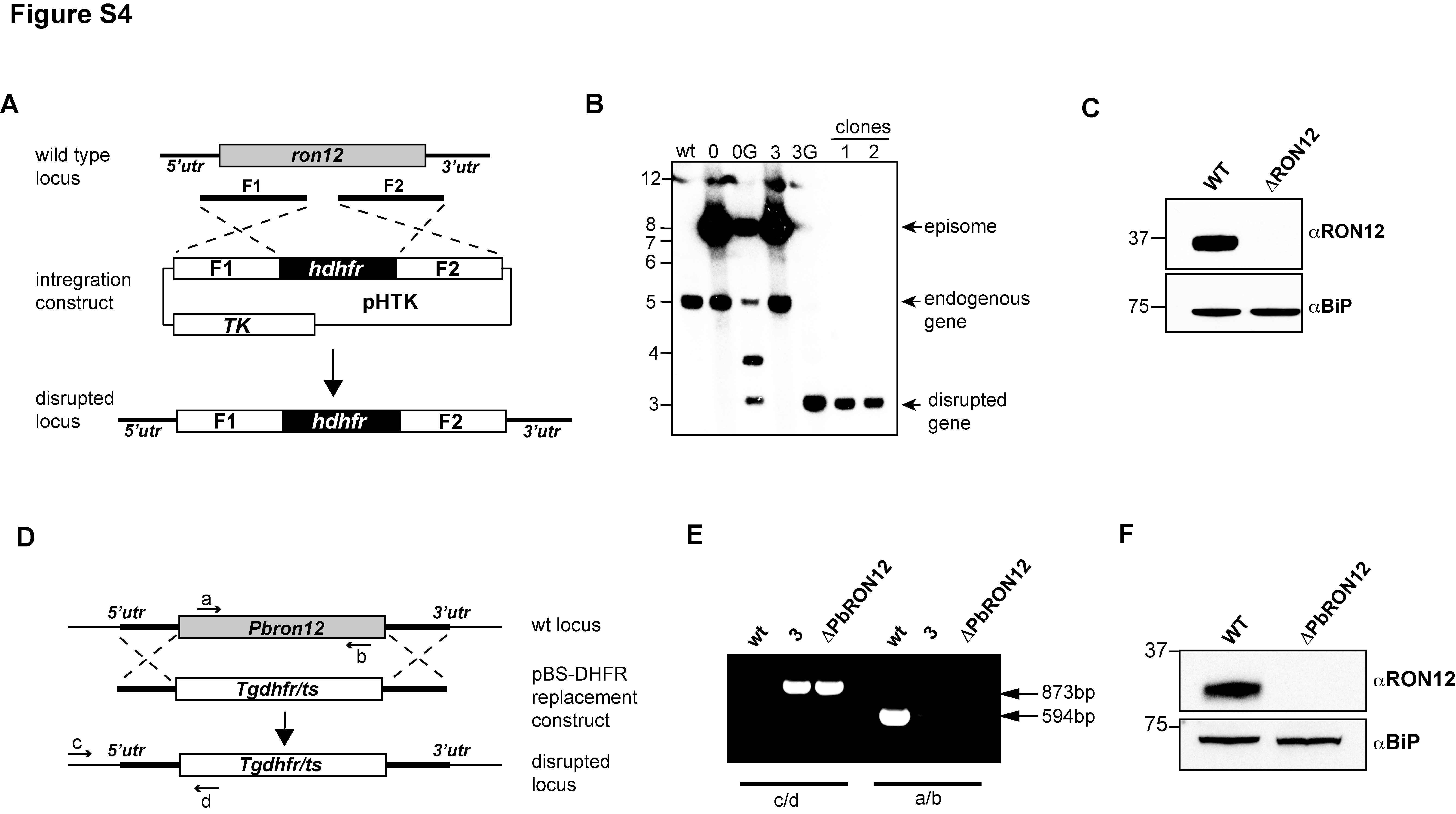

Supplement: Supplementary file 4 [file cmi0016-0657-SD4.tif]

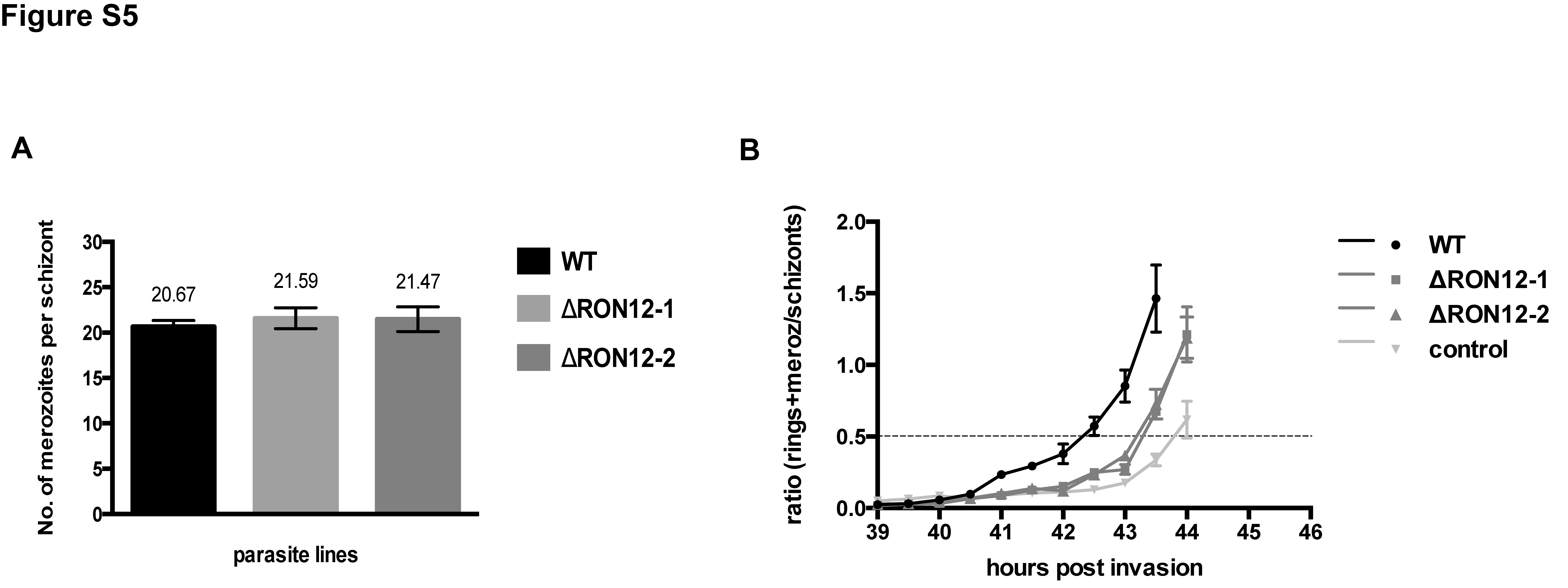

Supplement: Supplementary file 5 [file cmi0016-0657-SD5.tif]

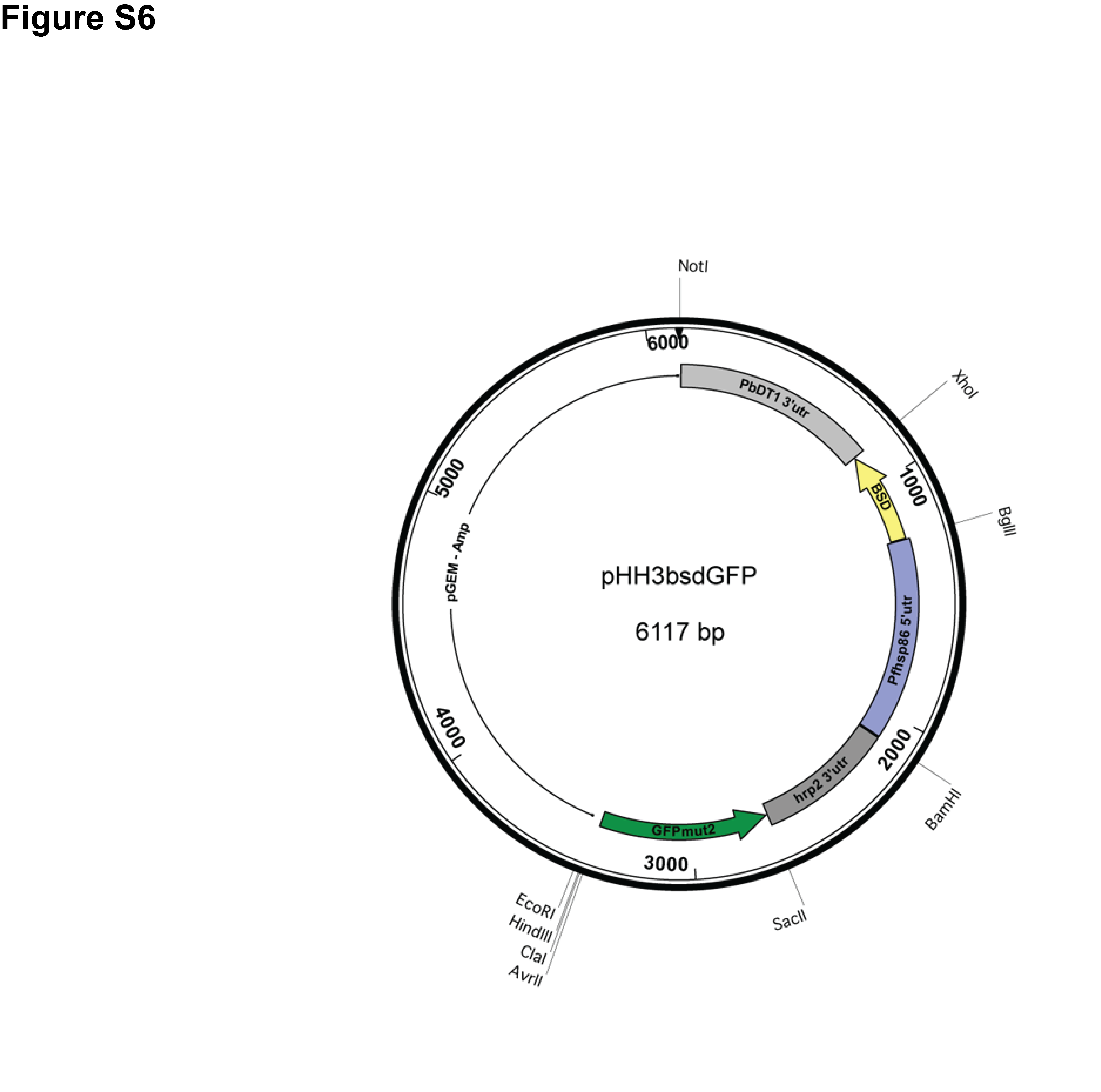

Supplement: Supplementary file 6 [file cmi0016-0657-SD6.tif]
